# Determination of Branched-Chain Amino Acids in Food Supplements and Human Plasma by a CE-MS/MS Method with Enhanced Resolution

**DOI:** 10.3390/ijms22158261

**Published:** 2021-07-31

**Authors:** Juraj Piestansky, Michaela Matuskova, Ivana Cizmarova, Dominika Olesova, Peter Mikus

**Affiliations:** 1 Department of Pharmaceutical Analysis and Nuclear Pharmacy, Faculty of Pharmacy, Comenius University, Odbojarov 10, SK-832 32 Bratislava, Slovakia; matuskova53@uniba.sk (M.M.); svorcova7@uniba.sk (I.C.); 2 Toxicologic and Antidoping Center, Faculty of Pharmacy, Comenius University, Odbojarov 10, SK-832 32 Bratislava, Slovakia; 3 Institute of Neuroimmunology, Slovak Academy of Science, Dubravska cesta 9, SK-845 10 Bratislava, Slovakia; dominika.olesova@savba.sk

**Keywords:** capillary electrophoresis, mass spectrometry, branched chain amino acids, food supplements, plasma, bioanalysis

## Abstract

In the presented study, a capillary electrophoresis-mass spectrometry method combining high separation efficiency and sensitive detection has been developed and validated, for the first time, to quantify branched chain amino acids (valine, isoleucine, leucine) in commercial food and sport supplement samples and human plasma samples. The separations were performed in a bare fused silica capillary. The background electrolyte was composed of 500 mM formic acid with pH 2.0. The plasma sample pretreatment was realized by simple protein precipitation with acetonitrile. Injection of a short zone of highly basic electrolyte before the sample injection and application of the negative pressure on the separation were accompanied by enhanced resolution of the isobaric amino acids—isoleucine and leucine. The developed method was characterized by favorable validation parameters, such as linearity (r^2^ > 0.99), accuracy and precision, the limit of detection, lower limit of quantification, or robustness. These parameters were more than sufficient for the quantification of branched chain amino acids in various samples. The determined concentrations of branched chain amino acids in food and sports supplements were in very good agreement with the content declared by the manufacturer. The investigated concentrations of branched chain amino acids were in the range 294.68–359.24 µM for valine, 91.76–95.67 µM for isoleucine, and 196.78–251.24 µM for leucine. These concentrations fall within the physiological limits. The developed CE-MS/MS method represents a suitable alternative to traditional approaches used in branched chain amino acid quality control and bioanalysis.

## 1. Introduction

**Figure 1:**
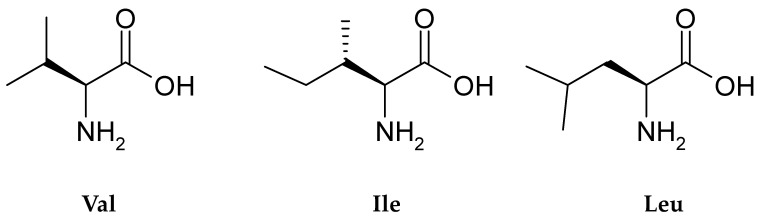
Chemical structures of branched-chain amino acids (BCAA)—valine (Val), isoleucine (Ile), and leucine (Leu).

Branched-chain amino acids (BCAAs) represent a group of three essential proteinogenic amino acids—valine, leucine, and isoleucine (Figure 1). The BCAAs are associated with the synthesis of proteins and energy production, where they act as substrates [1,2]. Their role in enhancing muscle protein synthesis and mass during exercise training [3,4], in the syndrome of cachexia [5], and aging [6,7] has been reported. Moreover, it was demonstrated that leucine is responsible for the activation of the anabolic signaling molecule—mammalian target of rapamycin complex 1 (mTORC1) [8]. Besides the involvement in protein metabolism, BCAAs also affect neurotransmission and glucose metabolism [9]. In recent years, several various novel functions of BCAAs associated with the gastrointestinal tract, immune system, mitochondrial biogenesis, or oxidative stress have been described [10]. Moreover, novel associations between BCAAs and chronic human diseases have been reported recently [11]. This is due to an increased number of metabolomic studies and the growing implementation of metabolomics in human disease research.

Metabolomics is a scientific field, usually focused on the identification and/or determination of a broad range of metabolites (non-targeted metabolic profiling) or a limited number of specific metabolites with a high level of chemical similarity (targeted analysis) [12]. The crucial methodologies used in metabolomics are mainly based on a combination of liquid chromatography (LC) or gas chromatography (GC) with mass spectrometry (MS). Furthermore, capillary electrophoresis (CE) hyphenated with MS represents a very attractive separation tool for metabolomic analysis [13,14], because many of the metabolites in biological systems are charged and the CE is a separation technique that is well-suited for such compounds. Nevertheless, in comparison with other analytical techniques, the use of CE-MS is still underestimated [15].

CE-MS represents a very useful analytical method for amino acid analysis that can be used for the investigation of various types of samples. In comparison with other more convenient strategies, the growing interest in this analytical tool is supported by some excellent review papers [16,17,18]. The CE-MS approach combines benefits such as separation of amino acids in their native form (without the need for any excessive derivatization procedure) according to their charge-to-size ratio with highly selective and sensitive detection offering unequivocal identification and quantification of the demanded analytes present in a complex biological matrix. Moreover, CE can be characterized as a method of “green analytical chemistry”, particularly considering the aqueous separation environment, very low consumption of organic solvents, and small sample size. Among other benefits are high separation efficiency, precision comparable to the LC, ease of automation, simplicity, high sample throughput due to the relatively short analysis time, low cost, and the fact that there is little or no sample pretreatment necessary [19]. 

In CE, amino acids can be separated as cations or anions depending on the composition of the background electrolyte—BGE [20]. The selection of an appropriate separation environment in the case of the CE-MS online combination is limited by the demands on volatility and low ion strength. Therefore, small molecular organic acids, such as formic (HFo) or acetic (HAc) acid, are the preferred BGE constituents. This fact is supported by some previously published papers dealing with CE-MS analysis of proteinogenic amino acids, especially in biological fluids [21,22,23,24,25,26,27,28,29] or with CE analysis in combination with conductivity detection focused on BCAAs only [30,31].

The reported CE-MS strategies are typically based on a targeted metabolomic approach that covers a large number of analytes—metabolites. Except for one study, the baseline separation between isobaric compounds such as isoleucine and leucine usually was not obtained [25]. The absence of appropriate resolution between the analytes may cause problems with the exact quantification of the compounds and, finally, it may lead to misinterpretation of the measured data. A fine-tuning of the separation conditions and working parameters is therefore necessary. Some papers have presented improvement in the resolution between the aforementioned isobaric amino acids [23], but the baseline separation was not obtained. 

The CE-MS quantification of BCAAs is usually performed in biological fluids; however, this analytical strategy could also be effectively implemented in the analysis of food supplements and pharmaceutical quality control. To the best of our knowledge, no study has been conducted and published dealing with such an advanced approach in this area. The quality control of a nutritional supplement containing BCAAs has been performed only with the use of capillary zone electrophoresis [32] or micellar electrokinetic chromatography (MEKC) with UV detection [33,34]. However, the demand for accurate and highly reliable analytical methods capable of ensuring and proving the high quality of food supplements or pharmaceuticals is still a subject of interest. 

The aim of this work was the development of a simple CE-MS method for precise determination of BCAAs in various types of samples characterized by complex matrices (i.e., pharmaceutical, food, and biological samples) that could be used in quality control and bioanalytical laboratories. Different separation and working conditions were examined to achieve the appropriate resolution of the analytes from each other, the optimum selectivity, and the MS detection response. The developed analytical procedure was carried out after the validation procedure and was successfully applied for the determination of BCAAs in an energy drink, sport nutritional supplement, and human plasma. Such a broad application range unequivocally confirms the increasing importance of the CE methods in various fields of industry, medicine, and science. 

## 2. Results and Discussion

### 2.1. Optimization

#### 2.1.1. Optimization of the Background Electrolyte (BGE)

**Figure 2:**
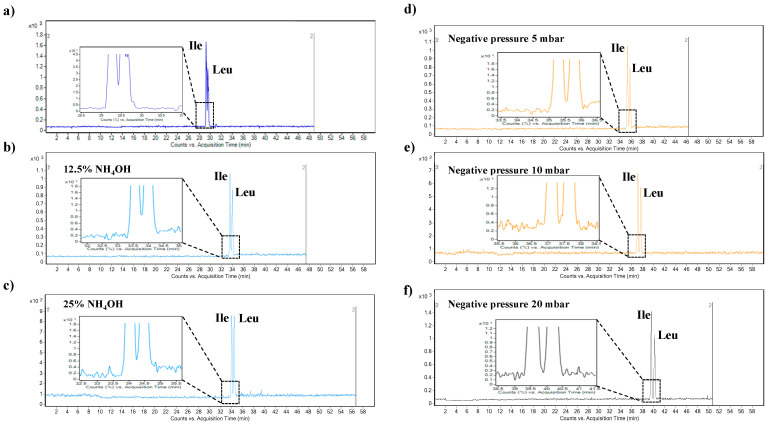
Effect of the NH_4_OH short plug injection before the sample injection and effect of the negative pressure application on the separation of isobaric amino acids isoleucine (Ile) and leucine (Leu). (**a**) Extracted multiple reaction monitoring (MRM) record of Ile and Leu obtained under convenient separation conditions. (**b**) Extracted MRM record of Ile and Leu obtained from the injection of a 12.5% NH_4_OH solution before the sample injection. The NH_4_OH solution was injected for 5 s. (**c**) Extracted MRM record of Ile and Leu obtained under convenient separation conditions and an injection of a 25% NH_4_OH solution before the sample injection. The NH_4_OH solution was injected for 5 s. (**d**) Extracted MRM record of Ile and Leu obtained from the injection of a 12.5% NH_4_OH solution before the sample injection, and application of negative pressure at the value 5 mbar. (**e**) Extracted MRM record of Ile and Leu obtained from the injection of a 12.5% NH_4_OH solution before the sample injection, and application of negative pressure at the value 10 mbar. (**f**) Extracted MRM record of Ile and Leu obtained from the injection of a 12.5% NH_4_OH solution before the sample injection, and application of negative pressure at the value 20 mbar. The concentration of the injected sample was 25 µM. For details of the capillary electrophoresis-tandem mass spectrometry method, see Section 3.

The composition of the BGE plays a crucial role in successful CE method development. In the case of CE-MS methods, the selection of suitable electrolytes is limited by the compatibility with the MS detection step. Here, the BGE optimization process was performed with the use of HFo in the concentration range of 100–1000 mM. Our results show that higher HFo concentrations were associated with enhanced resolution between the selected amino acids and with increased time of analysis, which is in agreement with the results presented in our previous work [28]. Nevertheless, the baseline separation between leucine and isoleucine was not achieved (see Figure 2a). The concentration of 500 mM (pH 2.0) was selected for further optimization of the method because no additional benefit from increased HFo concentration was obtained. The 500 mM HFo was able to offer appropriate separation of the BCAAs from the matrix constituents and good sensitivity capable of analyzing BCAAs at low concentrations in food supplements and plasma samples.

Furthermore, we investigated the effect of the injection of a short plug of highly basic electrolyte (here, ammonium hydroxide—NH_4_OH) before the sample injection. This approach is typically used as a simple in-capillary preconcentration method. It is well suited for amphiprotic analytes—e.g., amino acids [35]. The effect of NH_4_OH concentration (12.5% and 25%) and the time of hydrodynamic injection (in the range 5–15 s) was tested. In terms of signal intensity and signal-to-noise (S/N) ratio, the best results were obtained with the use of 12.5% NH_4_OH and its hydrodynamic introduction by applying a pressure of 20 mbar for 5 s. This approach was also characterized by enhanced resolution of isobaric amino acids Ile and Leu. Longer injection times of NH_4_OH were accompanied by increased migration times of the BCAAs and increased resolution, especially between Val and Ile. However, no significant increase of resolution between Ile and Leu was observed. Similarly, no significant benefit regarding the analytical signal intensity (expressed as S/N ratio) was observed. These findings are demonstrated by the data summarized in Table 1. The effect of the NH_4_OH concentration injected as a short plug into the separation capillary before the sample on the separation of isobaric amino acids—Ile and Leu—is presented in Figure 2a–c. According to the presented data, injection of 12.5% NH_4_OH for 5 s before the sample injection was applied in further experiments.

#### 2.1.2. Effect of the Negative Pressure on the Separation of BCAAs

The conventional CE-MS configuration is equipped with an electrospray (ESI) interface that utilizes a triple tube sprayer. Some papers dealing with the detailed optimization procedures of the ESI working parameters have proven the generation of a hydrodynamic flow inside the separation capillary due to a suction effect at the capillary tip [36,37,38,39,40]. It is an unwanted phenomenon because of its responsibility for extra zone broadening and decreased separation efficiency. Extremely detailed and very precise optimization of ESI parameters such as nebulizing gas flow rate and drying gas temperature represent one possibility to overcome this effect [41]. The second one involves the application of negative pressure at the inlet vial [42,43]. The effect of negative pressure on the separation of BCAA was investigated in our work. Application of negative pressure was accompanied by increased migration time, increased separation efficiency (see Table 2), and enhanced resolution between isobaric compounds Ile and Leu. The effect on Ile-Leu resolution is demonstrated in Figure 2d–f. Moreover, comparable reproducibility of the migration times was obtained with the method using the application of negative pressure (see RSD_tm_ in Table 2). According to the excellent separation parameters, further analysis was performed with the application of negative pressure at the 20 mbar level.

#### 2.1.3. Optimization of the Sheath Liquid

The sheath liquid composition is a crucial parameter in the CE-MS optimization procedure due to its impact on the magnitude of the analytical signal and chemical noise, and the resulting sensitivity of the method. It is typically composed of an organic and water phase with the addition of volatile acids and/or their ammonia salts. This provides high compatibility with MS, generation of a stable electrospray, and satisfactory/reliable ionization of the analytes. Two types of sheath liquids were tested in this work. The first one was composed of 0.1% HFo (which corresponds to 0.03 mM HFo) in 50% methanol, and the second one was represented by a mixture of 5 mM ammonium acetate (NH_4_Ac) and methanol at the ratio of 50/50 (*v*/*v*). As shown in Table 3, the optimal results were obtained using the sheath liquid composed of 5 mM NH_4_Ac in 50% methanol. The addition of volatile ammonia salt was not only beneficial in terms of enhanced resolution between the isobaric compounds; it also offered the highest analytical signal value and acceptable levels of chemical noise, hence resulting in favorable signal-to-noise (S/N) ratio.

Subsequently, it was necessary to investigate the effect of sheath liquid flow rate, which can also affect the S/N ratio and thus the sensitivity of the method. This is mainly due to the dilution effect on the separated zones during the electrospray ionization in the ESI interface. The tested values of the flow rate were in the range of 2–10 µL/min. Finally, a flow rate of 8 µL/min was selected as optimal as it offered an appropriate S/N ratio, stability of the analytical signal, and generation of a stable electrospray. 

#### 2.1.4. Optimization of the Mass Spectrometer Parameters

In this step, MS parameters such as capillary voltage, nebulizing gas pressure, drying gas temperature, and drying gas flow rate were investigated. Optimization of these parameters is necessary to obtain satisfactory detection sensitivity. Settings that offered the highest signal intensity and stability were selected. The optimum values were: capillary voltage 4 kV (tested range 3–5.5 kV), nebulizing gas pressure 10 psi (tested range 5–20 psi), drying gas temperature 300 °C (tested range 150–350 °C), and drying gas flow rate 10 L/min (tested range 3–10 L/min).

To further enhance selectivity and sensitivity, fragmentation behavior was characterized, using different triple quadrupole (QqQ) software operation modes—Scan mode, Selected ion monitoring (SIM), Product ion mode, and multiple reaction monitoring (MRM). In this process, we identified characteristic parent and product ion m/z values, fragmentor voltage, and collision energy—parameters important for the unequivocal determination of BCAAs. The characteristic values for each BCAA are summarized in Table 4.

#### 2.1.5. Optimization of the Plasma Sample Preparation

The use of different organic solvents (methanol, isopropanol, and acetonitrile) for protein precipitation was investigated to find the best protocol for the preparation of plasma samples. These organic solvents were added to a defined amount of plasma in a ratio of 3:1. Pooled plasma was used in these experiments. After the precipitation and centrifugation, supernatants were directly injected into the CE apparatus. The obtained results are summarized in Table 5.

**Figure 3:**
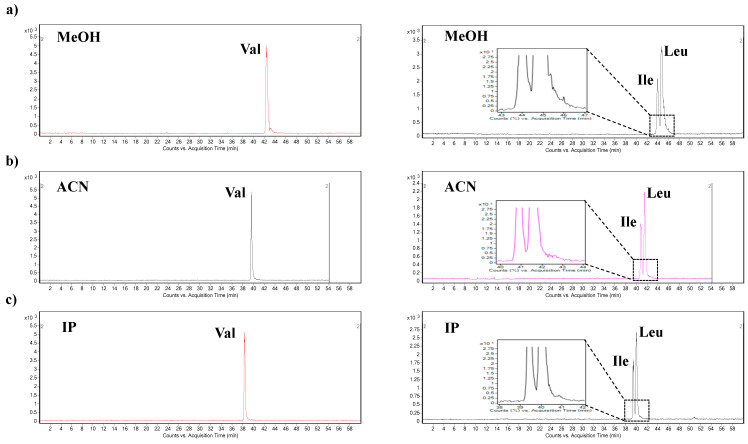
Optimization of the protein precipitation procedure for plasma samples. (**a**) Extracted MRM records of Val (left trace) and Ile and Leu (right trace), obtained after the plasma sample precipitation procedure with methanol (MeOH). (**b**) Extracted MRM records of Val (left trace) and Ile and Leu (right trace), obtained after the plasma sample precipitation procedure with acetonitrile (ACN). (**c**) Extracted MRM records of Val (left trace) and Ile and Leu (right trace), obtained after the plasma sample precipitation procedure with isopropanol (IP). For details of the capillary electrophoresis-tandem mass spectrometry method, see Section 3.

Illustrative electropherograms obtained from the precipitation agent testing are presented in Figure 3. The use of methanol as the precipitation agent was accompanied by a significant decrease of the S/N ratios and poor peak shapes, which resulted in separation efficiency deterioration (Figure 3a). The use of isopropanol was characterized by improved S/N ratio and resolution parameters. Similar results were obtained with the use of acetonitrile (Figure 3b,c). During the plasma sample preparation step, the recovery of the extraction procedure was investigated for each analyte. The recoveries were obtained by spiking the plasma samples with BCAAs standard at 25 µM concentration level. The highest recoveries (≥88%) of BCAAs were obtained with the use of acetonitrile as a precipitation agent. Finally, acetonitrile was selected as the most suitable precipitation agent for further experiments and validation procedures.

### 2.2. Validation

The optimized CE-MS method was validated according to the ICH Q2(R1) guideline [44] and the US Food and Drug Administration (FDA) guidance for bioanalytical method validation [45]. The ICH Q2(R1) guideline was used for the validation procedure focused on food and sport supplement samples. The FDA guideline was used for the validation procedure in the analysis of biological samples (plasma). Calibration parameters and some important performance parameters obtained for analytical standards either in pure water or spiked in plasma are summarized in Table 6. The linearity exceeded one decadic order for all three BCAAs with a determination coefficient (r^2^) higher than 0.996. The developed method was characterized by high separation efficiency (~200,000 theoretical plates) and appropriate limit of detection (LOD) and lower limit of quantification (LLOQ) values (~2 µM, and 5 µM, respectively). These parameters were more than suitable for quantification purposes of the BCAAs in various matrices (food, pharmaceutics, and biological liquid). Further, the developed method was characterized by excellent selectivity that enabled the resolution of the BCAAs from each other.

Specificity is one of the crucial parameters investigated during the validation procedure. It proves the ability to assess unequivocally the analyte in the presence of various expected matrix components. The specificity of the developed and optimized CE-MS method was proved. Owing to the use of tandem mass spectrometry (MS/MS) detection, which enables one to work with a very selective multiple reaction monitoring (MRM) regime, there were no interfering components present in migration positions of monitored BCAAs.

To support the implementation of the developed CE-MS method into the field of food and sports supplement analysis, parameters such as accuracy, precision, and robustness were investigated according to the ICH Q2(R1) validation guideline. Accuracy and precision were tested by the analysis of seven concentration levels in the range 5–500 µM. The intra-day precision was determined by investigating the samples within one day. The inter-day precision was evaluated by repeated analysis of the samples in three replicates per day for 5 days. According to the data summarized in Table 7, we can conclude that the developed method is sufficiently precise—the RSD values of determined BCAAs concentrations were below 7%. Moreover, the presented data reflected the appropriate accuracy of the tested method—the calculated relative error (RE%) for each BCAA was lower than 15%, which is in good agreement with the ICH guideline acceptance criteria. 

The robustness of an analytical method is defined as a measure of its capacity to obtain comparable and acceptable results after carrying out small but deliberate variations in procedural parameters. Here, the tested factors were variation in BGE composition (±1 mM) and variation in pH of the BGE (±0.1). These small but deliberate variations of the operation parameters resulted in fluctuations of the migration time and peak area of all tested BCAAs of less than 1.5% and 2.3%, respectively. We can conclude that the developed method is robust and can be reliably used under standard conditions without any unwanted effect. Results from the validation procedure show that the developed CE-MS method is suitable for its application in the field of BCAAs quality control.

A similar validation procedure was used for the evaluation of the biomedical method. Here, the recommendations according to the FDA guideline were applied. The validation procedure of the bioanalytical method is typically performed with the use of specific quality control (QC) samples. The QC samples are prepared by spiking the same matrix as the study sample (here, plasma) with the tested analytes at three concentration levels—low, medium, and high. 

The accuracy and precision results obtained from the analysis of QC samples at the three concentration levels (low—25 µM, medium—100 µM, high—300 µM) are summarized in Table 8. The intra-day accuracies for all concentration levels and all analytes were 92.9–104.9%, while the inter-day accuracies were in the range 95.4–104.3%. The intra- and inter-day precision were not higher than 5.9% and 7.1%, respectively. The obtained results were within the FDA acceptance criteria (±15%).

Two types of stability experiments were performed with the QC plasma samples to investigate the impact of various storage conditions on the BCAAs. Results from short-term (storage in autosampler for 24 h) and freeze-to-thaw (three freeze-thaw cycles when the samples were frozen and subsequently thawed at laboratory temperature) stability testing are summarized in Table 9. The observed relative error did not exceed 10.2%, which indicates acceptable stability of the BCAAs (FDA criteria ±15%). It can be stated that the proposed plasma sample handling and preparation procedures are suitable for routine use.

**Figure 4:**
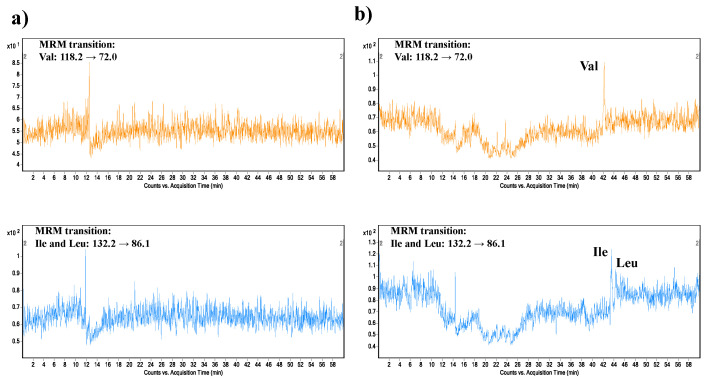
Extracted MRM records of Val (upper trace) and Ile and Leu (lower trace), obtained from the carryover experiment. (**a**) Analysis of BCAAs in blank sample injected after the analysis of plasma QC sample at medium concentration level (100 µM). (**b**) Analysis of BCAAs in blank sample injected after the analysis of plasma QC sample at high concentration level (300 µM). For details of the capillary electrophoresis-tandem mass spectrometry method, see Section 3.

Carryover represents the appearance of the analyte in a sample from a preceding sample. It is a critical parameter that affects the reliable quantitation of the study samples. The FDA guideline does not provide any description of the carryover testing procedure; however, the acceptance criteria are defined—carryover should not exceed 20% of LLOQ. To investigate the carryover parameter, we injected blank water samples after each QC sample concentration level. No presence of BCAA peaks in electropherograms was observed after the analysis of samples containing the analytes at QC low and QC medium concentration levels (Figure 4a). In the case of the analysis performed after the sample at QC high concentration level, the carryover for Val responded to 1.7% LLOQ, Ile 1.5% LLOQ, and Leu 0.1% LLOQ (Figure 4b). The obtained results fulfill the FDA acceptance criteria. It can be suggested that the negligible carryover effect was obtained due to the integrated preconditioning step of the separation capillary. This procedure represents an application of a voltage with opposite polarity to the run voltage. It is a simple and well-documented procedure that is recommended to improve the precision of CE measurements [46].

### 2.3. Application

**Figure 5:**
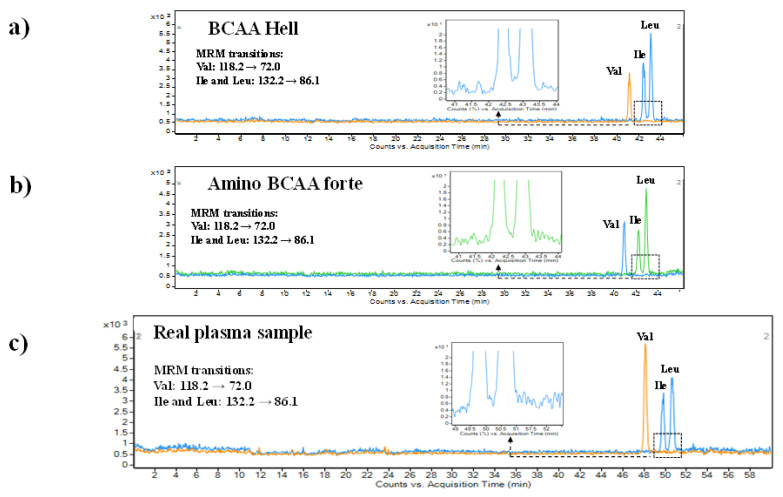
Extracted MRM records of Val, Ile, and Leu were obtained from the analysis of BCAA in an energy drink (**a**), sport supplement (**b**), and real human plasma sample (**c**). For details of the capillary electrophoresis-tandem mass spectrometry method, see Section 3.

The developed and validated CE-MS/MS method was finally applied to determine the content of BCAAs in food and sport supplements samples, and real human plasma samples. In comparison to plasma, the food and sport supplements samples are characterized by less complex matrix constituents. However, the presence of other various active ingredients (e.g., presence of vitamins, caffeine, or taurine in energy drinks) or pharmaceutical excipients (e.g., fillers, binders, lubricants, taste modifiers, etc.) can affect the analysis of demanded substances. The use of highly selective MS detection and the application of the MRM detection regime was favorable in term of obtaining records that were not affected by the presence of other substances present in the real samples. This fact is documented by the records obtained from the analysis of the samples, which are presented in Figure 5a,b. Moreover, it was also demonstrated that no extensive sample preparation for analysis was necessary. Only a simple dilution procedure was suitable to obtain highly reproducible and reliable results. This fact is supported by the recovery data, which were evaluated by spiking the real samples with BCAAs standards. The recovery values for the three analytes were—97.7%, Val; 97.4%, Ile; and 99.0%, Leu. The determined concentrations of BCAAs in an energy drink (Hell Active) and capsules (BCAA Active) are summarized in Table 10. The obtained concentrations were in very good agreement with the data declared by the manufacturer. The determined concentrations were in the range of 94–106% of the declared concentrations. 

In comparison to previously mentioned food and sports supplements, the analysis of plasma samples is more challenging. This is due to the fact that it is represented by a complex multi-component matrix that contains a large scale of heterogeneous substances. The main problems are typically associated with the presence of a relatively high amount of proteins. Direct injection of plasma samples into CE is not recommended because the proteins may easily precipitate in the separation capillary with a small internal diameter. Hence, this can lead to clogging of the separation capillary and frequent breakages of current. This finally results, not only in disruption of the separation procedure, but also in the destruction of the separation capillary and to the need for its frequent exchange. Therefore, it was necessary to remove the majority of proteins present in the plasma sample. The optimization procedure proved that acetonitrile was a suitable and most effective protein precipitation agent. A simple protein precipitation procedure followed by direct injection of the supernatant into the CE apparatus was used in the case of BCAAs analysis in real human plasma samples. The effectiveness of the sample pretreatment, the high separation efficiency of the CE, and high selectivity and sensitivity of the MS detection can be documented by the BCAAs records presented in Figure 5c. As can be seen, the baseline separation between isobaric compounds (Ile and Leu) was preserved. The BCAAs concentrations determined for the two healthy volunteers plasma samples are summarized in Table 11. The obtained BCAAs levels in plasma are consistent with the levels of these compounds reported for normal specimens in a large number of studies, which are summarized in a complex database—Human Metabolome Database [47]. The recovery percentages of the three analytes that were spiked in the real plasma samples were 89% for Val, 91.3% for Ile, and 92.4% for Leu. According to the presented findings, it can be stated that the developed CE-MS/MS method is suitable for the analysis of various sample types characterized by different levels of complexity. Moreover, it was demonstrated that precise method development and optimization of some critical factors of the CE-MS hyphenation may lead to reliable analyses, which are highly demanded and valued in the biomedical environment.

### 2.4. Comparison of Methods for BCAAs Analysis

Table 12 provides a summary of some convenient approaches for BCAAs (or all amino acids) analysis based on chromatographic and electrophoretic methods. The traditional methods used in amino acid analysis (including BCAAs) are based on liquid chromatography (LC) in combination with UV or fluorescence detection. The ion exchange chromatography (IEX) with postcolumn ninhydrin derivatization is considered as a gold standard method for amino acid analysis and is used for diagnostic purposes of some metabolic disorders. The main drawback of this traditional LC method is accompanied by extremely long times of analysis, which can take approx. 3 h [48]. Recent trends in amino acid (including BCAAs) analysis are focused on LC-MS/MS methods, which are characterized by short time of analysis, high throughput, and LODs for BCAAs in the range of 0.1–2.5 µM [49,50,51,52,53]. However, some of these methods suffer from poor resolution between isobaric BCAAs Ile and Leu, which affects the precision of the method (inter-day RSD > 15%) [53].

Popular approaches for BCAAs analysis in plasma are based on reversed-phase high performance liquid chromatography (HPLC) using precolumn derivatization. Here, the total time of analysis takes from 30 to 60 min, and the LOD values varies from 2 to 24 µM [54,55]. Moreover, precolumn derivatization is costly and time-consuming and it requires controlled conditions to achieve appropriate robustness, reliability, and reproducibility.

Other analytical strategies for BCAAs determination are based on gas chromatography (GC) in combination with mass spectrometry. However, extensive derivatization procedures are required. The sample preparation is time consuming and expensive. Moreover, this process can be associated with the loss of the sample and formation of various byproducts, which can finally lead to misinterpretation of the generated data. Another issue is the time of analysis, which can be more than 1 h [56].

The CE methods used in analysis of BCAAs are typically hyphenated with MS detection [22,25,57,58], but some papers refer to the use of UV [32], laser induced fluorescence (LIF) [58], or capacitively coupled contactless conductivity detection (C^4^D) [30]. The CE-LIF method required conversion of the analytes into their fluorescently active derivatives using an expensive off-line derivatization procedure [58]. The CE-UV methods (direct and indirect UV detection) used for determination of BCAAs in pharmaceutical sample suffered from poor resolution between Ile and Leu (resolution was in the range 0.8 to 1.0). Moreover, there was a need to implement various BGE additives into the separation environment (e.g., cyclodextrines), which made the optimization procedure more challenging [32]. The published CE-MS methods were characterized by time of analysis in the range of 20–40 min and LOD values from 0.6 to 200 µM. Better LOD values of BCAAs were obtained for matrices with lower protein content, such as urine [22,25].

**Table 12:** Overview of the chromatographic and electrophoretic methods used for determination of BCAAs in various sample types.

Method	Derivatization	Matrix	Sample Preparation	t (min)	LOD (µM)	Ref.
IEX-UV	ninhydrin	plasma	Protein precipitation	~180	-	[48]
LC-MS/MS	-	plasma	Protein precipitation	10	-	
LC-MS/MS	-	dried blood spot	Extraction methanol/water	15	-	[49]
LC-MS/MS	-	Serum	Protein precipitation	2	0.1–0.8	[50]
LC-MS/MS	-	plasma	Protein precipitation	8	0.5–0.6	[51]
LC-MS/MS	-	plasma	Protein precipitation	10	0.5–2.5	[52]
LC-MS/MS	-	plant leaves	Extraction	75	0.3	[53]
HPLC-fluorescence	OPD	plasma	Protein precipitation	30	22.4–24.3	[54]
HPLC-fluorescence	AQC	plasma	Protein precipitation	53	>2	[55]
GC-MS	N-methyl-N-(trimethylsilyl)fluoroacetamide	serum	Extraction, protein precipitation	>60	-	[56]
CE-MS	-	soy sauce	Dilution	~20	1.3–3.4	[57]
CE-MS/MS	-	urine	Dilution	~25	0.6–1.0	[22]
CE-MS	-	urine	Dilution	~30	-	[25]
CE-MS	-	blood	Protein precipitation	40	9–200	[59]
CE-direct UV	-	pharmaceutical	Hydrolysis	20	24.6–37.2	[32]
CE-indirect UV	-			13	7.3–11.8	
MEKC-UV	FMOC-Cl	pharmaceutical	Dilution	40	4.8	[33]
CE-LIF	NBD-F	cell culture	Microdialysis	0.5	0.1–0.4	[58]
CE-C^4^D	-	plasma	Protein precipitation	~2	0.4	[30]
CE-MS/MS	-	plasma, beverage, pharmaceutical	Dilution, Protein precipitation	~45	0.7–2.49	Recent study

AQC—6-aminoquinolyl-N-hydroxysuccinimidyl carbamate, C^4^D—capacitively coupled contactless conductivity detection, FMOC-Cl—9-Fluorenylmethoxycarbonyl chloride, IEX—ion exchange chromatography, NBD-F—4-fluoro-7-nitrobenzofurazan, OPD—O-phenylenediamine.

In comparison to previously published methods for BCAAs analysis in model and biological samples, our CE-MS/MS methods offers the possibility to perform the analysis without the need of a derivatization step or other extensive sample pretreatment steps. The sample preparation is rapid as no derivatization, extraction, or drying steps are involved. The analysis of biological samples (here, plasma) requires only simple deproteinization by the addition of acetonitrile. The consumption of the plasma sample required for the analysis is very low—only 10 µL. The obtained LODs are comparable with the modern LC-MS/MS or fast CE approaches combined with fluorescence or C^4^D detection. Moreover, the presented LOD values obtained by our approach were improved more than 80 times in comparison to previously published CE-MS method for analysis of plasma samples [59]. 

The presented data support the fact that our CE-MS/MS represents a valuable alternative to traditionally used chromatographic (GC, HPLC) or modern LC-MS/MS methods. This analytical approach has been demonstrated to be a sensitive and environmentally friendly method that can be advantageously used as a reference as well as a routine method in laboratories focused on the quality control and/or bioanalysis of BCAAs.

## 3. Materials and Methods

### 3.1. Chemicals and Samples

The commercially available amino acid standard mixture in 0.1 M HCl, which contains L-valine, L-leucine, and L-isoleucine, was obtained from Sigma–Aldrich (Steinheim, Germany). The concentration of the demanded amino acids in the standard mixture was 2.5 mM. The LC-MS grade chemicals used for the preparation of the BGE and sheath liquid solution were purchased from Merck (Darmstadt, Germany), Sigma–Aldrich (Steinheim, Germany), and VWR International (Vienna, Austria). Ultra-pure CE (Agilent Technologies, Santa Clara, CA, USA) was used as a solvent for the electrolytes, sheath liquid, and samples. The electrolyte solutions were filtered before use through disposable 0.22 µm pore size membrane filters (Millipore, Molsheim, France) and were stored in the fridge before their use. An energy drink, Hell Active (Hell Energy, Szikszó, Hungary), and a food supplement (capsule) Amino BCAA Forte (Kompava, Nové Mesto nad Váhom, Slovakia) were obtained from a local store. 

### 3.2. Instrumentation

The electrophoretic measurements were carried out using the Agilent 7100 CE system (Agilent Technologies, Santa Clara, CA, USA) coupled to an Agilent 6410 Series Triple Quadrupole (Agilent Technologies, Santa Clara, CA, USA) mass spectrometer using a co-axial sheath-liquid CE-MS interface. A sheath liquid composed of 5 mM NH_4_Ac in 50% (*v*/*v*) methanol water was delivered through an Agilent 1260 Infinity isocratic pump (Agilent Technologies, Santa Clara, CA, USA) at a flow rate of 8 µL/min. The electrospray (ESI) was operated in positive mode. The mass spectrometer settings comprise nebulizing gas (nitrogen) pressure, 10 psi; drying gas temperature, 300 °C; drying gas flow rate, 10 L/min; capillary voltage, 5000 V; and dwell time, 50 ms. An Agilent ChemStation B.04.03 software and Mass Hunter Work Station B.03.01 software (both from Agilent Technologies, Santa Clara, CA, USA) were used for CE and MS data acquisition and system control. 

Uncoated fused silica capillaries with 50 µm inner diameter (id) and 120 cm total length (MicroSolv Technology Corporation, Eatontown, NJ, USA) were used in all experiments. Each new capillary was conditioned by applying a pressure flush of 950 mbar (standard internally generated pressure) for at least 30 min with 1 M NaOH, followed by ultra-pure CE water and BGE. At the end of each workday, the capillary was rinsed with 0.1 M NaOH for 10 min, ultra-pure CE water for 20 min, and BGE for 10 min and stored in BGE overnight. The samples were injected into the capillary hydrodynamically with a pressure of 50 mbar for 20 s. Before the sample injection, a short plug of 12.5% NH_4_OH was introduced into the capillary hydrodynamically by applying a pressure of 50 mbar for 5 s. Separation was performed at a voltage of +25 kV, which was gradually increased at the beginning of the separation from 0 to +25 kV for 18 s. During the separation procedure, a backpressure of 20 mbar was applied. To reduce the carry-over, before each injection, the capillary was re-equilibrated by applying negative voltage, −20 kV, for 30 s and flushing with BGE for 2 min. 

### 3.3. Standard Solution and Sample Preparation

#### 3.3.1. Standard Solutions, Calibration Solutions, and Quality Control (QC) Samples

The stock solution of analytical standards was prepared by appropriate dilution of the amino acid standards with 0.1 M HCl, obtaining 500 µM concentration levels. Working solutions (i.e., calibration and QC) were made by a proper dilution of the stock solution with ultra-pure CE water or by spiking the human plasma samples with the stock solution. Each sample was measured in six replicates.

Calibration standards were prepared by dilution with ultra-pure CE water to obtain the following concentrations: 5, 15, 30, 60, 125, 250, and 500 µM. Each point of the calibration line was measured in six replicates.

The QC samples were prepared at three concentration levels, i.e., 25 µM (low), 100 µM (medium), and 300 µM (high) by spiking the pooled plasma samples with the standard solution. A 10 µL aliquot of each QC sample was transferred to an Eppendorf tube followed by 30 µL of an appropriate organic solvent. After 20 min at laboratory temperature, the samples were centrifuged at 13,000× *g* for 10 min. The supernatant was then transferred to a CE vial and directly analyzed by the CE-MS method. Each sample was measured in three replicates.

#### 3.3.2. Food Supplement Sample Preparation

Two types of food supplements were analyzed in this work. The beverage (energy drink Hell Active) sample was prepared as follows: 100 mL of the beverage was transferred to the volumetric flask and sonicated for 30 min. The solution was then filtered using Whatman filter paper No.1. From the filtrate, a 1.0 mL portion of the beverage was pipetted into a 100 mL volumetric flask and diluted with the ultra-pure CE water. The solution was then directly analyzed by the CE-MS method. The sample was measured in three replicates.

The commercial sports nutritional supplement Amino BCAA Forte (capsules) containing 192 mg Leu, 92 mg Ile, and 92 mg Val per capsule, was prepared in a very similar manner. The powder content of five capsules of this preparation was individually weighed and homogenized into a fine mixture. An amount of powder equivalent to the weight of one capsule (400 mg) was transferred to a 1000 mL volumetric flask using about 50 mL 0.1 M HCl. The solution was sonicated for 30 min. Dilution was made up to the mark with ultra-pure CE water, and the mixture was then filtered using Whatman filter paper No.1. A 1.0 mL portion of the filtrate was transferred into a 100 mL volumetric flask and diluted with ultra-pure CE water. The solution was then directly analyzed by the CE-MS method. The sample was measured in three replicates. 

**Figure 6:**
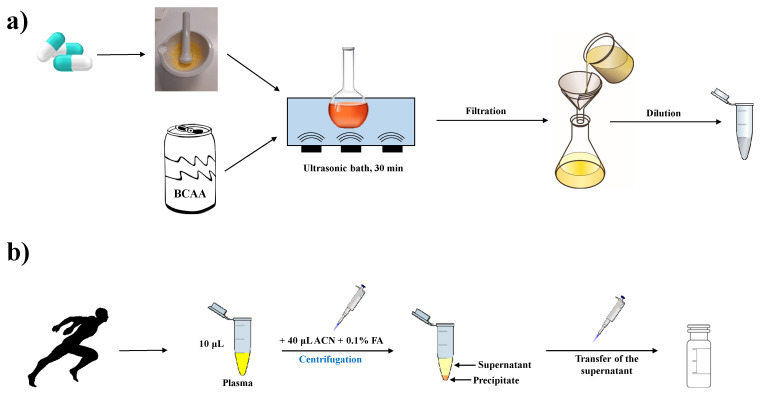
Sample preparation workflow—(**a**) preparation of food and sports nutritional supplements, (**b**) preparation of human plasma samples.

The whole procedure of the food and sports supplement sample preparation before the CE-MS analysis is graphically illustrated in Figure 6a. 

#### 3.3.3. Plasma Sample Collection and Preparation

Real human plasma samples were obtained from two healthy male volunteers. Fasting blood samples were collected in the morning in test tubes containing EDTA. Plasma was obtained by centrifugation (12,000× *g*, 10 min.) within 30 min of sample collection. The samples were then aliquoted and stored at −20 °C until further analysis. Before analysis, the plasma samples were left to thaw at 4 °C, and then 10 µL of each sample was transferred to an Eppendorf tube and 30 µL of acetonitrile was added to precipitate proteins. After 20 min at laboratory temperature, the samples were centrifuged at 13,000× *g* for 10 min. The supernatant was then transferred to a CE vial and directly analyzed by the CE-MS method. Each sample was measured in three replicates. A simplified scheme of the plasma sample preparation for the CE-MS analysis is presented in Figure 6b.

## 4. Conclusions

A CE-MS/MS method was, for the first time, developed and validated for the quantitation of BCAAs in food, sports supplements, and human plasma samples. The efficiency of the developed method was sufficient for baseline separation of the isobaric analytes—leucine and isoleucine. This effect was obtained owing to the fine-tuning of some separation and detection parameters. The benefits of the proposed method also include the fact that there is no need for derivatization of the samples and there is the possibility to analyze BCAAs in their naïve form, simple sample pretreatment (dilution—food and sport supplements samples, protein precipitation—plasma samples), favorable performance parameters, and exceptional reliability. Moreover, our work represents the first in-depth study dealing with the investigation of various factors that may affect separation efficiency, the intensity of the analytical signal, and resolution of the analytes (e.g., injection of short plug of highly basic solution before the sample plug, application of negative pressure during the analysis).

The complex validation realized in the presented study proved that the method can be effectively applied, not only in the quality control of food and sports supplements, but also in the biomedical environment. It was demonstrated that a precise analytical development led to a highly reliable CE-MS method suitable for the bioanalysis of BCAAs.

## Figures and Tables

**Table 1:** Optimization of the NH_4_OH concentration and time of its hydrodynamic injection into the separation capillary.

	Without NH_4_OH				12.5% NH_4_OH				25% NH_4_OH			
	Val	Ile		Leu	Val	Ile		Leu	Val	Ile		Leu
t_m_ (min)	29.81	30.37		30.70	32.76	33.57		34.00	33.19	34.02		34.47
RSD_tm_ (%)	0.6	0.6		0.7	0.5	0.6		0.5	1.7	1.9		2.0
S/N	30.4	19.5		12.7	63.9	32.3		31.8	55.2	31		24.2
N	110,500	126,400		97,600	168,200	192,700		177,400	174,500	170,300		177,100
R	2.28		1.18		2.63		1.36		2.57		1.38	
	**5 s**				**10 s**				**15 s**			
	**Val**	**Ile**		**Leu**	**Val**	**Ile**		**Leu**	**Val**	**Ile**		**Leu**
t_m_ (min)	32.76	33.57		34.00	36.12	37.11		37.62	38.52	39.57		40.14
RSD_tm_ (%)	0.5	0.6		0.5	0.6	0.8		0.8	0.9	1.1		1.1
S/N	63.9	32.3		31.8	63.6	31.0		30.8	59.3	31.5		30.5
N	168,200	192,700		177,400	268,700	204,800		170,000	270,700	200,800		180,200
R	2.63		1.36		3.29		1.37		3.35		1.39	

S/N—signal-to-noise ratio, R—resolution, N—separation efficiency. Resolution (R) was calculated according to the equation: R = 1.18 * (t_2_ − t_1_)/(w_1/2 (A)_ + w_1/2 (B)_), where t_2_ is the migration time of the analyte with lower electrophoretic mobility, t_1_ is the migration time of the analyte with higher electrophoretic mobility, w_1/2 (A)_ is the full width at half maximum of the peak migrating in the time t_1_, and w_1/2 (B)_ is the full width at half maximum of the peak migrating in the time t_2_. The concentration of each BCAA in the injected sample was 25 µM.

**Table 2:** Effect of the negative pressure application on the separation of BCAA.

	No pressure				5 mbar				10 mbar				20 mbar			
	Val	Ile		Leu	Val	Ile		Leu	Val	Ile		Leu	Val	Ile		Leu
t_m_ (min)	32.76	33.57		34.00	33.95	34.79		35.41	36.06	37.09		37.66	39.80	40.96		41.65
RSD_tm_ (%)	0.5	0.6		0.5	0.8	0.8		0.8	0.8	0.7		0.8	0.9	0.7		0.8
S/N	57.9	31.0		24.2	60.1	34.0		31.0	64.5	37.7		35.8	65.8	42.7		40.6
N	168,200	192,700		177,400	174,000	184,100		183,700	190,400	184,900		206,600	243,100	192,000		214,800
R	2.63		1.36		2.68		1.46		3.08		1.69		3.18		2.13	

S/N—signal-to-noise ratio, R—resolution, N—separation efficiency. Resolution (R) was calculated according to the equation: R = 1.18 * (t_2_ − t_1_)/(w_1/2 (A)_ + w_1/2 (B)_), where t_2_ is the migration time of the analyte with lower electrophoretic mobility, t_1_ is the migration time of the analyte with higher electrophoretic mobility, w_1/2 (A)_ is the full width at half maximum of the peak migrating in the time t_1_, and w_1/2 (B)_ is the full width at half maximum of the peak migrating in the time t_2_.

**Table 3:** Effect of the sheath liquid composition on BCAA separation.

	0.1% HFo * in Methanol				5 mM NH_4_Ac in Methanol			
	Val	Ile		Leu	Val	Ile		Leu
t_m_ (min)	34.59	35.42		35.89	38.44	39.57		40.14
S/N	35.8	29.1		21.4	59.3	54.5		40.0
N	159,000	180,000		170,800	270,700	207,700		200,400
R	2.76		1.44		4.15		1.99	

* 0.1% HFo corresponds to 0.03 mM HFo. S/N—signal-to-noise ratio, R—resolution, N—separation efficiency. Resolution (R) was calculated according to the equation: R = 1.18 * (t_2_ − t_1_)/(w_1/2 (A)_ + w_1/2 (B)_), where t_2_ is the migration time of the analyte with lower electrophoretic mobility, t_1_ is the migration time of the analyte with higher electrophoretic mobility, w_1/2 (A)_ is the full width at half maximum of the peak migrating in the time t_1_, and w_1/2 (B)_ is the full width at half maximum of the peak migrating in the time t_2_.

**Table 4:** Selected parent-product ion m/z transitions and MS conditions used for BCAAs quantification.

	Parent Ion (*m*/*z*)	Product Ion (*m*/*z*)	Fragmentor (V)	Collision Energy (eV)
Val	118.2	72.0	100	10
Leu	132.2	86.1	100	12
Ile	132.2	86.1	100	12

**Table 5:** Effect of the precipitation agent on the plasma sample preparation for BCAA analysis.

	Methanol				Isopropanol				Acetonitrile			
	Val	Ile		Leu	Val	Ile		Leu	Val	Ile		Leu
t_m_ (min)	42.36	43.95		44.71	39.62	40.86		41.51	38.27	39.45		40.04
S/N	151.9	42.0		63.8	413.5	84.1		132.3	413.1	66.3		96.7
N	78,900	101,300		66,500	162,500	134,000		105,000	142,000	194,000		144,000
R	2.75		1.21		3.28		1.36		3.09		1.52	
Recovery (%)	77.8	85.3		76.8	78.3	80.1		79.8	88.0	88.3		89.4

S/N—signal-to-noise ratio, R—resolution, N—separation efficiency. Resolution (R) was calculated according to the equation: R = 1.18 * (t_2_ − t_1_)/(w_1/2 (A)_ + w_1/2 (B)_), where t_2_ is the migration time of the analyte with lower electrophoretic mobility, t_1_ is the migration time of the analyte with higher electrophoretic mobility, w_1/2 (A)_ is the full width at half maximum of the peak migrating in the time t_1_, and w_1/2 (B)_ is the full width at half maximum of the peak migrating in the time t_2_.

**Table 6:** Selected operation and validation parameters of the CE-MS/MS method.

		Water			Plasma	
	Valine	Isoleucine	Leucine	Valine	Isoleucine	Leucine
t_m_ (min)	38.52	39.57	40.14	40.77	41.92	42.58
RSD_tm_ (%), *n* = 6	0.34	0.46	0.33	2.93	3.00	3.02
RSD_area_ (%), *n* = 6	4.02	1.32	2.26	3.75	0.63	2.13
a (counts)	−767.24	−22.388	−32.00	3451.7	3071.2	385.76
RSD_a_ (%), *n* = 6	7.34	3.31	3.00	1.71	1.25	3.78
b (counts × µM^−1^)	163.96	30.829	188.09	390.15	429.31	408.88
RSD_b_ (%), *n* = 6	1.40	0.83	1.12	5.35	4.91	4.81
r^2^	0.9982	0.9994	0.9990	0.9964	0.9969	0.9982
Linear range (µM)	5–500	5–500	5–500	5–500	5–500	5–500
LOD (µM)	0.70	1.33	1.57	2.04	2.27	2.49
LLOQ (µM)	1.2	2.5	2.5	5	5	5
N	349,505	398,753	346,512	213,973	240,049	190,192

LOD and LLOQ were calculated as the signal (S) to noise (N) ratios to be 3 × S/N and 5 × S/N, respectively. Separation efficiency (N) was calculated according to the equation N = 5.545 * (t_m_/w_1/2_)^2^, where w_1/2_ is the full width at half the maximum of the peak. The calibration curve is expressed by the equation y = b.x + a. RSD_tm_ and RSD_area_ were calculated from the samples at the LLOQ concentration level.

**Table 7:** Accuracy and precision of the CE-MS method investigated according to the ICH Q2(R1) guideline.

	Nominal (µM)	Found(µM)			RSD (%)			RE (%)		
		Val	Ile	Leu	Val	Ile	Leu	Val	Ile	Leu
Intra-day, *n* = 5	5	5.27	4.86	4.62	6.8	6.7	5.6	5.4	−2.8	−7.6
	15	15.14	14.04	14.98	4.9	6.6	3.4	0.9	−6.4	−0.1
	30	29.17	27.07	28.98	5.2	6.3	4.3	−2.8	−9.8	−3.4
	60	62.84	58.89	61.44	5.8	2.9	6.1	4.7	−1.9	2.4
	125	132.22	123.86	127.69	4.0	0.7	2.1	5.8	−1.0	2.2
	250	253.22	255.07	245.89	3.4	4.5	4.8	1.3	2.0	−1.6
	500	503.69	508.89	506.01	1.9	4.3	3.5	0.7	1.8	1.2
Inter-day, *n* = 15	5	4.81	4.46	4.29	12.2	6.0	4.4	−3.8	−10.9	−14.3
	15	14.20	14.08	14.48	5.0	3.1	6.7	−5.3	−6.1	−3.5
	30	28.51	28.75	30.18	3.4	7.6	1.1	−5.0	−4.2	0.6
	60	63.94	64.61	56.75	2.4	5.6	6.0	6.6	7.7	−5.4
	125	122.13	122.37	121.74	4.2	0.5	9.9	−2.3	−2.1	−2.6
	250	239.03	252.24	236.93	2.8	3.1	2.3	−4.4	0.8	−5.2
	500	516.06	499.32	505.77	3.3	1.7	3.4	3.2	−0.1	1.2

**Table 8:** Accuracy and precision data of the QC samples.

	Intra-Day, *n* = 5			Inter-Day, *n* = 15		
	Valine	Isoleucine	Leucine	Valine	Isoleucine	Leucine
	QC low					
Nominal (µM)	25	25	25	25	25	25
Found (µM)	25.58	26.22	23.23	25.39	24.66	23.86
Accuracy (%)	102.3	104.9	92.9	101.6	98.6	95.4
RSD (%)	2.3	5.7	4.3	2.6	3.9	4.9
	QC medium					
Nominal (µM)	100	100	100	100	100	100
Found (µM)	98.49	102.38	105.92	97.58	96.14	104.29
Accuracy (%)	98.5	102.4	105.9	97.6	96.1	104.3
RSD (%)	1.9	4.0	4.4	2.2	2.9	4.2
	QC high					
Nominal (µM)	300	300	300	300	300	300
Found (µM)	284.43	297.30	304.25	302.57	304.84	310.20
Accuracy (%)	94.8	99.1	101.4	100.9	101.6	103.4
RSD (%)	4.6	5.4	5.9	6.1	7.1	6.4

**Table 9:** Stability testing of valine, leucine, and isoleucine in QC samples.

	Autosampler Stability			Freeze-to-Thaw Stability		
	Valine	Isoleucine	Leucine	Valine	Isoleucine	Leucine
	QC low, *n* = 5					
Nominal (µM)	25	25	25	25	25	25
Found (µM)	24.77	24.22	27.55	26.06	24.01	26.12
Accuracy (RE%)	−0.9	−3.1	10.2	4.2	−4.0	4.4
	QC medium, *n* = 5					
Nominal (µM)	100	100	100	100	100	100
Found (µM)	95.26	95.61	97.22	98.13	92.87	97.72
Accuracy (RE%)	−4.7	−4.4	−2.8	−1.9	−7.1	−2.3
	QC high, *n* = 5					
Nominal (µM)	300	300	300	300	300	300
Found (µM)	296.76	302.01	297.35	290.05	307.93	294.71
Accuracy (RE%)	−1.1	0.7	−0.9	−3.3	2.6	−1.8

**Table 10:** Determined concentrations of BCAAs in tested food samples.

	Valine			Isoleucine			Leucine		
	Declared (mg/mL)	Found (mg/mL)	RSD (%), *n* = 3	Declared (mg/mL)	Found (mg/mL)	RSD (%), *n* = 3	Declared (mg/mL)	Found (mg/mL)	RSD (%), *n* = 3
Hell Active	0.090	0.088	2.5	0.090	0.085	2.7	0.180	0.173	2.2
BCAA Active	0.092	0.098	4.1	0.092	0.090	2.9	0.192	0.204	3.1

**Table 11:** Determined concentrations of BCAAs in tested plasma samples.

	Valine		Isoleucine		Leucine	
	c (µM)	RSD (%)	c (µM)	RSD (%)	c (µM)	RSD (%)
Plasma vol.1	359.24	7.5	91.76	5.2	196.78	7.0
Plasma vol.2	294.68	6.9	95.67	6.4	251.24	6.8

## Data Availability

Data is contained within the article.
